# Initial Esophageal Anastomosis Diameter Predicts Treatment Outcomes in Esophageal Atresia Patients With a High Risk for Stricture Development

**DOI:** 10.3389/fped.2021.710363

**Published:** 2021-09-07

**Authors:** Osama Baghdadi, Susannah Clark, Peter Ngo, Jessica Yasuda, Steven Staffa, Benjamin Zendejas, Thomas Hamilton, Russell Jennings, Michael Manfredi

**Affiliations:** ^1^Division of Gastroenterology, Hepatology and Nutrition, Boston Children's Hospital, Boston, MA, United States; ^2^Department of Surgery, Boston Children's Hospital, Boston, MA, United States; ^3^Department of Anesthesiology, Critical Care and Pain Medicine, Boston Children's Hospital, Boston, MA, United States

**Keywords:** endoscopy, esophageal atresia, anastomotic strictures, pediatrics gastroenterology, esophagus, esophageal diameter, esophageal dilatation, esophageal balloon

## Abstract

**Background and Aims:** Children with esophageal atresia (EA) who undergo surgical repair are at risk for anastomotic stricture, which may need multiple dilations or surgical resection if the stricture proves refractory to endoscopic therapy. To date, no studies have assessed the predictive value of anastomotic diameter on long-term treatment outcomes. Our aim was to evaluate the relationship between anastomotic diameter in the early postoperative period and need for frequent dilations and stricture resection within 1 year of surgical repair.

**Methods:** A retrospective chart review was performed of patients who had EA repair or stricture resection (SR). Medical records were reviewed to evaluate the diameter of the anastomosis at the first endoscopy after surgery, number and timing of dilations needed to treat the anastomotic stricture, and need for stricture resection. A generalized estimating equations (GEE) modeling with a logit link and binomial family was done to analyze the relationship between initial endoscopic anastomosis diameter and the outcome of needing a stricture resection. Median regression was implemented to estimate the association between number of dilations needed based on initial diameter.

**Results:** A total of 121 patients (56 females) with a history of EA (64% long-gap EA) were identified who either underwent Foker repair at 46% or stricture resection with end-to-end esophageal anastomosis at 54%. The first endoscopy occurred a median of 22 days after surgery. Among all cases, a narrower anastomoses were more likely to need stricture resection with an OR of 12.9 (95% CI, 3.52, 47; *p* < 0.001) in patients with an initial diameter of <3 mm. The number of dilations that patients underwent also decreased as anastomotic diameter increased. This observation showed a significant difference when comparing all diameter categories when looking at all surgeries taken as a whole (*p* < 0.008).

**Conclusion:** Initial anastomotic diameter as assessed *via* endoscopy performed after high-risk EA repair predicts which patients will require more esophageal dilations as well as the likelihood for stricture resection. This data may serve to stratify patients into different endoscopic treatment plans.

## Background

Children who undergo surgical repair of esophageal atresia (EA) are at risk for anastomotic stricture (AS) following surgical repair. Esophageal AS is one of the most common postoperative complications and occurs anywhere from 9 to 80% of EA patients (1–4). Esophageal AS can be treated with serial endoscopic dilation and adjunct therapies including steroid injections, incisional therapy, and stenting. However, treatment may require numerous dilations and may ultimately require surgical resection if the stricture proves refractory to therapy. Several risk factors have been reported for the development of an AS, including anastomotic leak, long-gap EA (LGEA), high-tension anastomosis, ischemic tissue ends, gastroesophageal reflux, and gestational age (5). To date, no evidence-based guidelines exist regarding screening children postoperatively for esophageal stricture. The recommended approach is endoscopy after a child exhibits symptoms of food and swallowing difficulties or failure to advance to a solid diet, at the appropriate age, after surgery (6). Also, there are no studies that have examined the relationship between anastomotic diameter assessed at time of initial postoperative endoscopy and treatment outcomes. This study examines the hypothesis that an anastomosis' initial diameter, when evaluated by endoscopy can predict the likelihood of requiring multiple AS dilations or require a stricture resection, in patients with risk factors for developing an AS.

## Methods

An institutional review board approved single-center retrospective chart review of patients with diagnosis of EA who underwent esophageal surgery and follow-up at our Esophageal and Airway Center between January 2016 and December 2019 was performed. Clinical data from patient charts particularly endoscopy/surgical and fluoroscopy reports were collected. Recorded patient information included type of EA, sex, gestational age, age at time of surgery, diagnosis of trisomy 21 and VACTERL association, number of days out from the surgery at the time of first endoscopy, initial anastomosis diameter, number of dilations in the first year after surgery, and stricture resection. LGEA was defined as any EA where the size of the gap length precluded the ability to complete a primary, one-stage surgical repair regardless of presence or absence of an associated tracheoesophageal fistula (TEF) (7–9).

It is our practice at the center that patients who have uncomplicated surgeries, non-LGEA with low anastomotic tension, and no leak or evidence of stricture on esophagram will be monitored for stricture based on clinical symptoms with repeat esophagram at ~6 months of age. Patients who do not meet these criteria are considered more high risk for AS and have endoscopy performed 3–4 weeks postrepair. If a stricture is identified, dilation is performed, and a series of additional planned endoscopies with possible dilation would be scheduled as needed (8). The development of AS after the Foker procedure and after stricture resection has been previously described (5, 9–11). High-risk AS patients in this study were divided into two groups, LGEA patients who underwent a Foker procedure, for tension-induced esophageal growth (12) and patients who had undergone a surgical stricture resection (SR) for a known AS refractory to endoscopic treatment. All patients in the SR group underwent a complete resection of their prior AS with the creation of a new end-to-end esophageal anastomosis. Patients who had a Heineke-Mikulicz stricturoplasty or other type of stricturoplasty were excluded.

**Figure 1:**
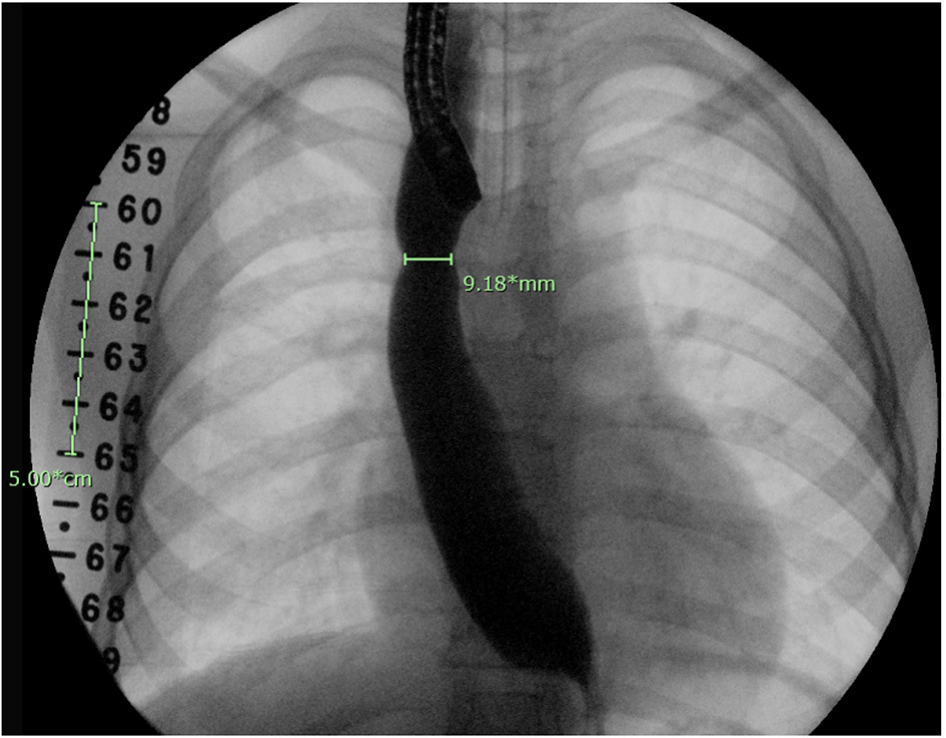
Esophagram done at time of endoscopy. A fluoroscopic ruler is seen on the right side of the patient as a reference for calibration.

**Figure 2:**
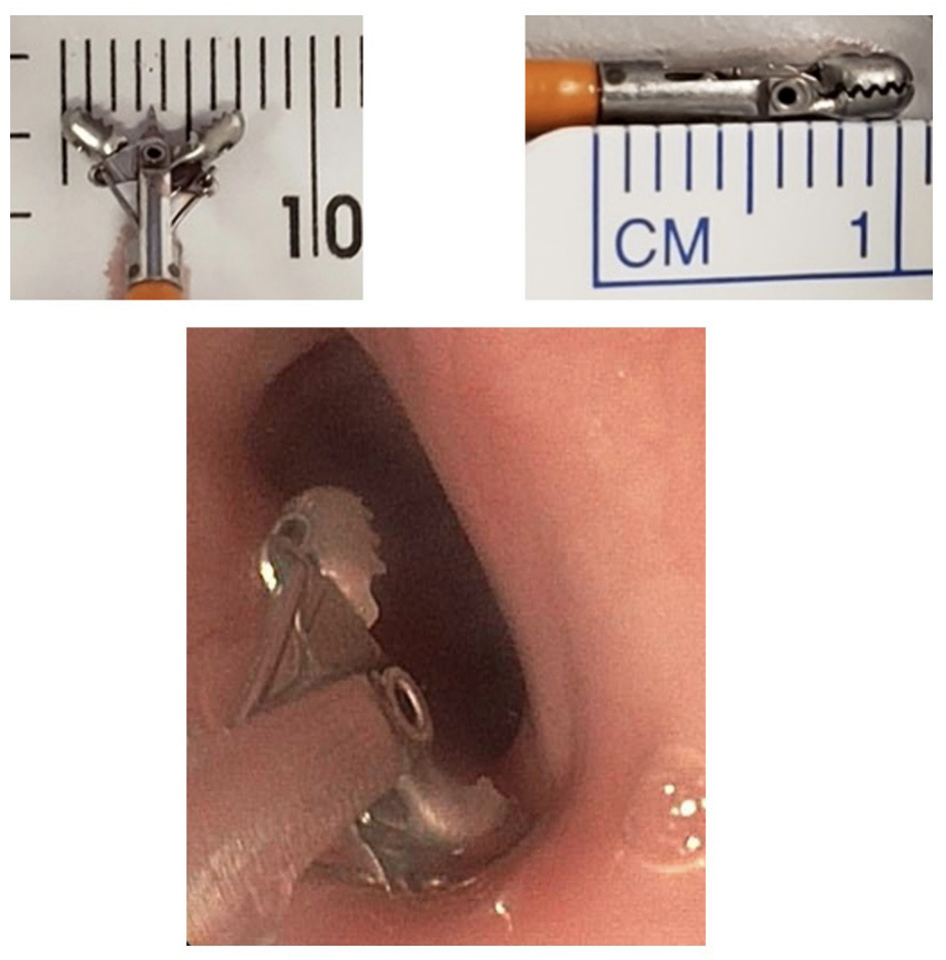
Reference measurement of the biopsy forceps open and closed.

The initial diameter of the esophagus was determined by contrast esophagram, performed during the first endoscopy following EA/stricture repair, with a radiopaque ruler placed under the patient (Figure 1). The anastomotic diameter was measured using the fluoroscopic image with the greatest anastomotic diameter; the radiopaque ruler and known endoscope diameter were used as size references. Additionally, the known width of open and closed biopsy forceps and known scope diameter were used to determine the diameter of the anastomosis in cases with poor contrast distention (Figure 2). All procedures were done by two experienced endoscopists that use similar techniques. The endoscopes used were either the Olympus XP190N or Olympus GIF 190 series. In each patient group, the AS diameter measurements were divided into the following subgroups for comparison: 0 to <3, 3 to <6, 6 to <9, and ≥9 mm. Patients were followed up for 1 year after surgical repair or until resolution of stricture seen on follow-up endoscopy or esophagram.

### Statistical Analysis

Demographics and patient characteristics were presented as median and interquartile range for continuous data and frequency and percentage for categorical data. The analysis of the relationship between anastomosis diameter at first endoscopy and the outcome of needing SR for refractory AS was performed using generalized estimating equations (GEE) modeling with a logit link and binomial family in order to account for multiple observations within the same patient. A two-tailed alpha level <0.05 was used to determine statistical significance, except for the analyses comparing between initial anastomosis diameter categories where a Bonferroni-adjusted *p* < 0.008 (0.05/6) was used to determine statistical significance to control for the risk of false-positive results (type I error) due to multiple group comparisons. All modeling results are presented using odds ratios (OR) with 95% confidence intervals (CI) and *p*-values. Stata (version 15.0, StataCorp LLC., College Station, TX, USA) was used to perform all statistical analyses.

Median regression was implemented to estimate the association between number of dilations needed and initial diameter, with results shown as coefficients with 95% confidence intervals and *p*-values.

## Results

### Demographics and Patients' Characteristics

We identified 121 patients with a history of EA who underwent a total of 141 surgeries (56 (46%) females, median age of 7 months (IQR, 4–14) at the time of surgical EA repair). There were 10 patients (8%) with Trisomy 21 and 25 (21%) with VACTERL association. From total surgeries, there were 65 (46%) Foker procedures for LGEA repair and 76 (54%) SR with end-to-end esophageal anastomosis. The first endoscopy occurred at a median of 22 days (IQR, 21–28) after surgery. Patients were noted to have three esophageal dilations (IQR, 2, 6) within 1 year following surgical repair (see Table 1 for reference).

**Table 1:** Demographic information.

**Demographic data**	
**Patients**	121
Female	56 (46%)
Gestational age (median weeks, IQR)	36 (33,38)
Age at surgery (median months, IQR)	7 (4, 14)
Trisomy 21	10 (8%)
VACTERL	25 (21%)
**Diagnosis**	
Long-gap esophageal atresia	78
Non-long-gap esophageal atresia	43
**Endoscopy**	
First endoscopy, postoperative day (median, IQR)	22 (21, 28)
Number of dilations 1 year from surgery (median, IQR)	3 (2, 6)
**Surgical repair**	141
Foker procedure (%)	65 (46%)
Stricture resection (%)	76 (54%)

*Data are presented as n (%) or median (interquartile range)*.

### Anastomotic Initial Endoscopic Diameter and Need for SR in All Surgeries

Looking at all surgeries combined (*N* = 141), 23 (16%) patients underwent a SR. A SR was more likely to occur in patients with a narrower initial diameter. The OR of requiring a SR was 12.9 (95% CI, 3.52, 47; *p* < 0.001) in patients with an initial diameter of <3 mm. When patients had a wider anastomosis diameter, 3 to <6 mm, the OR for requiring a SR decreased to 3.07 (95% CI, 0.97, 9.76; *p* = 0.056). Lastly, 25 cases had an initial diameter ≥9 mm, in which none underwent a SR (see Table 2 for reference).

**Table 2:** Analysis of need for stricture resection by anastomosis diameter at initial postoperative endoscopy among all cases and stratified by type of surgery.

**All surgeries (** ***N*** **= 141)**					
**Anastomosis diameter at first endoscopy**	**Needed stricture resection (** ***N*** **= 23)**	**Did not need stricture resection (** ***N*** **= 118)**	**Odds ratio**	**95% CI**	***p*** **-value**
0 to <3 mm (*N* = 16), *n* (row %)	9 (56%)	7 (44%)	12.9	(3.52, 47.0)	<0.001^*^
3 to <6 mm (*N* = 34), *n* (row %)	8 (24%)	26 (76%)	3.07	(0.97, 9.76)	0.056
6 to <9 mm (*N* = 66), *n* (row %)	6 (9%)	60 (91%)	Reference		
≥9 mm (*N* = 25), *n* (row %)	0 (0%)	25 (100%)	Omitted—no patients with stricture resection.		
**Foker (** ***N*** **= 65)**					
**Anastomosis diameter at first endoscopy**	**Needed stricture resection (** ***N*** **= 14)**	**Did not need stricture resection (** ***N*** **= 51)**	**Odds ratio**	**95% CI**	***p*** **-value**
0 to <3 mm (*N* = 12), *n* (row %)	6 (50%)	6 (50%)	24	(2.41, 238.9)	0.007^*^
3 to <6 mm (*N* = 24), *n* (row %)	7 (29%)	17 (71%)	9.88	(1.11, 87.9)	0.04^*^
6 to <9 mm (*N* = 25), *n* (row %)	1 (4%)	24 (96%)	Reference		
≥9 mm (*N* = 4), *n* (row %)	0 (0%)	4 (100%)	Omitted—no patients with stricture resection.		
**Stricture resection (** ***N*** **= 76)**					
**Anastomosis diameter at first endoscopy**	**Needed stricture resection (** ***N*** **= 9)**	**Did not need stricture resection (** ***N*** **= 67)**	**Odds ratio**	**95% CI**	***p*** **-value**
0 to <3 mm (*N* = 4), *n* (row %)	3 (75%)	1 (25%)	21.6	(1.87, 250.0)	0.014^*^
3 to <6 mm (*N* = 10), *n* (row %)	1 (10%)	9 (90%)	0.8	(0.08, 7.73)	0.847
6 to <9 mm (*N* = 41), *n* (row %)	5 (12%)	36 (88%)	Reference		
≥9 mm (*N* = 21), *n* (row %)	0 (0%)	21 (100%)	Omitted—no patients with stricture resection.		

*Odds ratios (OR), 95% confidence intervals, and p-values are derived from regression analysis. CI, confidence interval*.

* *Statistically significant. Astrix under the table defines the bolded values*.

### Anastomotic Initial Endoscopic Diameter and Need for SR Stratified by Type of Surgery

Sixty-five patients had undergone Foker repair for LGEA, and 14 (22%) underwent a stricture resection. When analyzing the diameter at initial endoscopy stratified by type of surgery, we noted a similar statistical pattern seen in the unstratified surgical group. The OR of requiring SR was 24 (95% CI, 2.41, 238.9; *p* = 0.007) in patients with a diameter of <3 mm. The OR decreased to 9.88 (95% CI, 1.11, 87.9; *p* = 0.04) when the diameter was wider measuring 3 to <6 mm. Four patients had an anastomotic diameter ≥9 mm, which did not require a SR (see Table 2 for reference).

Similarly, in the stricture resection group, patients (*N* = 76) with an initial diameter <3 mm on endoscopy had a 21.6 increased likelihood of another stricture resection (95% CI, 1.87, 250; *p* = 0.014). By comparison, no patients *N* = 21 with anastomosis ≥9 mm had another stricture. One patient (10%) required a stricture diameter with an anastomosis diameter of 3 to <6 mm (OR, 0.8; 95% CI, 0.08, 7.73; *p* = 0.847) resection (see Table 2 for reference).

### Anastomotic Initial Endoscopic Diameter and Need for Esophageal Dilation in the First Year

**Figure 3:**
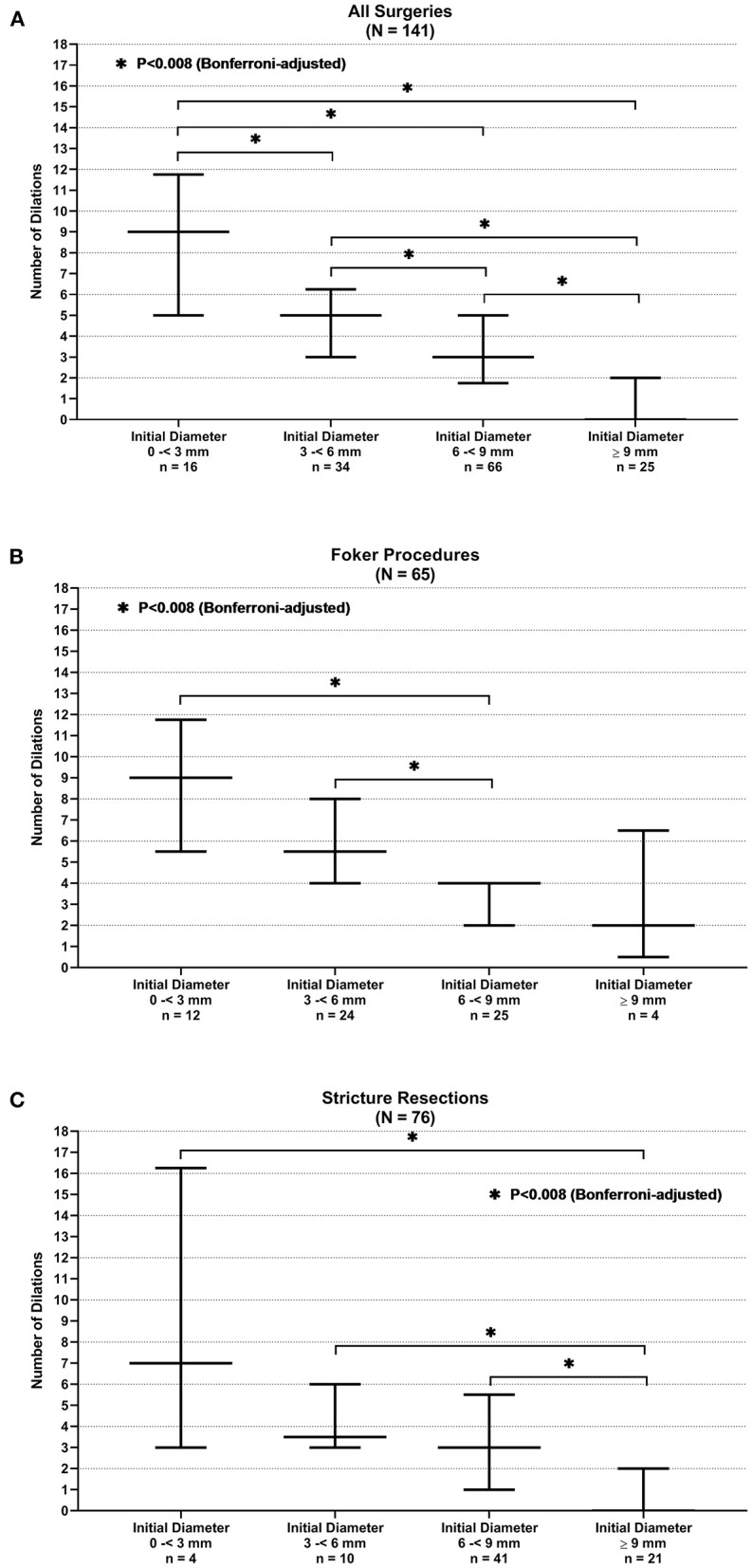
Anastomotic initial endoscopic diameter and need for stricture resection. **(A)** All surgeries. **(B)** Foker procedure. **(C)** Stricture resection.

**Figure 4:**
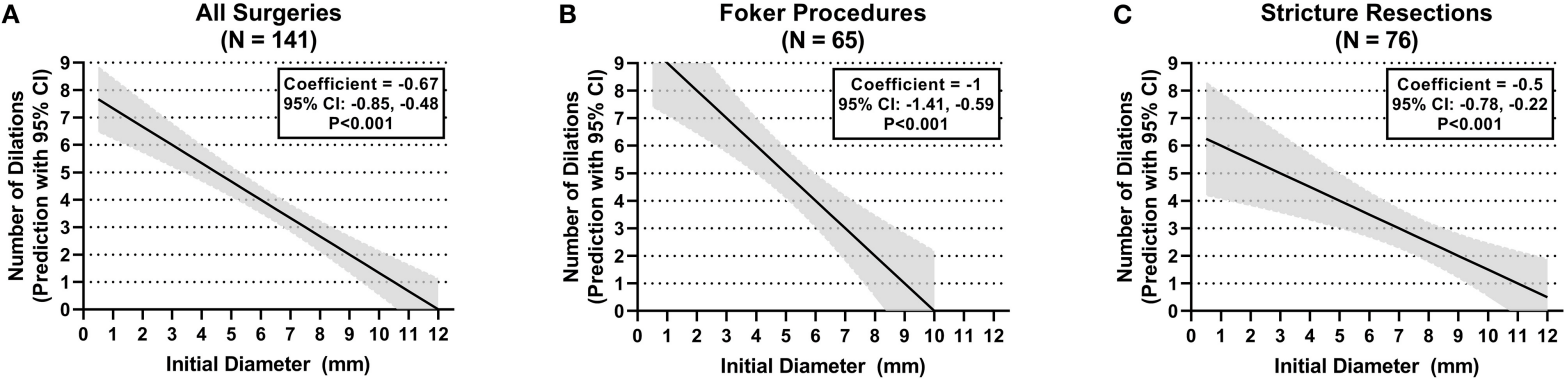
Anastomotic initial endoscopic diameter and need for dilations. **(A)** All surgeries. **(B)** Foker procedure. **(C)** Stricture resection.

The number of esophageal dilations that patients underwent decreased significantly as the initial anastomosis diameter increased in size seen on the first endoscopy following surgical repair. This observation showed a significant difference when comparing all diameter categories when looking at all surgeries taken as a whole (*p* < 0.008) (see Figure 3A). This was also illustrated in the median regression analysis. An increase in the initial diameter by 1 mm had coefficient of −0.67 dilations [95% CI, −0.85, −0.48; *p* < 0.001 (see Figure 4A)].

When stratifying surgeries, the Foker repair group reached statistically significant differences in the number of dilations when the initial diameter of 6 to <9 mm is compared with the initial diameters of <3 and 3–6 mm (*p* < 0.008) (see Figure 3B). A gain by 1 mm in initial diameter had a coefficient of −1 dilations (95% CI, −1.41, −0.59; *p* < 0.001) in the median regression analysis (see Figure 4B). However, the stricture resection group showed a statistically significant decrease in number of dilations between all initial diameter ranges when compared with an initial diameter ≥9 mm (*p* = 0.008) (see Figure 3C). Here, an increase by 1 mm in diameter had a coefficient of −0.5 dilations (95% CI, −0.78, −0.22; *p* < 0.001) (see Figure 4C).

## Discussion

This is the first study to look at anastomosis diameter, measured on initial endoscopic assessment after surgery in EA patients, as a predictor of future need for stricture resection and stricture dilations. In this study, the LGEA cohort of patients who has undergone a Foker procedure had the greatest need for stricture resection (22%) compared with patients in the stricture resection cohort (12%). In both cohorts of patients undergoing Foker procedure and in the stricture resection, there was a >20-fold increased likelihood of requiring a stricture resection if the initial diameter was ≤ 3 mm. This study also found an inverse relationship between the initial endoscopic anastomosis diameter and the number of dilations performed within 1-year postsurgical repair. Overall, the number of dilations significantly decreased as the initial diameter was wider.

The utility of risk stratification based on initial diameter may allow the provider to tailor a dilation schedule appropriate for each patient. In addition, it allows the provider to offer more information to patients and their families regarding the possible need for multiple dilations and the likelihood of a stricture resection in the future. This approach can be particularly useful for patients who are at high risk of developing an esophageal stricture. The authors acknowledge that this differs from the common approach of waiting for a patient to become symptomatic. Our study was not designed to evaluate the preferred approach to dilations in all EA patients; however, our data confirms that high-risk populations like those with LGEA or history of prior stricture resection are more likely to have anastomotic strictures that require multiple dilations. Therefore, a more proactive approach with early endoscopy may be considered in these populations. Clinically, esophageal stricture may cause vomiting, choking, dysphagia, and food impaction which may lead to oral aversion, which is one of the main causes of nutritional problems and is difficult to treat (13–15). It is a particular problem in children with EA; one study of 75 patients with EA found that 36% had a history of malnutrition and 54% were not taking age- or developmentally appropriate textures (14). The authors speculate that early effective detection and treatment of a stricture could help minimize feeding difficulties and oral aversion from developing.

Prior to this study, most attempts to predict outcomes of esophageal strictures utilized esophagram. Several studies have looked at esophageal measurements in different locations in order to create various stricture indexes to determine need for dilation in EA patients after surgery. These esophagrams were performed in the early postoperative period (5–10 days) (10, 16, 17). Only one of these found any statistical correlation between stricture indexes and any outcome (10); Landisch et al., in their 2017 study evaluating the efficacy of various stricture indexes in 45 EA patients, also evaluated this score and did not find it was significantly associated with need for dilation. The Landisch study did find esophagram measurements to be helpful when done farther out than the usual 5–10 days after surgery (18). These studies did not use the measurements to predict likelihood of stricture resection or assess median number of dilations based on the esophagram measurements. The Landisch study also suggests, as does our study, that the timing of the exam a month out from surgery may be what is the critical factor. Additionally, our results show that measurement of the anastomosis diameter alone without the need of a stricture index formula was useful to evaluate an anastomosis for increased risk of needing treatment.

Limitations of this study include the fact that it is a retrospective single-center experience with a large population of high-risk EA anastomoses. Our cohort was homogenous, including only pediatric EA patients, so our results may not be applicable to adults or to patients with strictures from other etiologies. We also acknowledge that determining the need for stricture resection is somewhat subjective with institutional bias. Prospective multicenter studies are needed to limit institutional bias. Furthermore, measurements of initial diameters are somewhat subjective, although the scopes that we use are of diameters similar to our groupings. In addition, we use fluoroscopy to confirm the diameter as an additional measure of accuracy, although this may not be available in all practice settings. We also feel having only two endoscopists who are making these estimations in a high-volume practice limits variability.

## Conclusion

This study finds that the initial endoscopic measurement of an esophageal anastomosis diameter is predictive for need of future stricture resection as well as the number of dilations that may be required to treat the anastomotic stricture. Postoperative endoscopic evaluations could serve to stratify patients into high- and low-risk groups, which allows for more tailored treatment plans and may help to better manage patient family expectations for likely course and outcome of treatment.

## Data Availability Statement

The raw data supporting the conclusions of this article will be made available by the authors, without undue reservation.

## Ethics Statement

The studies involving human participants were reviewed and approved by the institutional review board at Boston children's hospital. Written informed consent to participate in this study was provided by the participants or their legal guardian/next of kin.

## Author Contributions

All authors listed have made a substantial, direct and intellectual contribution to the work, and approved it for publication.

## Conflict of Interest

The authors declare that the research was conducted in the absence of any commercial or financial relationships that could be construed as a potential conflict of interest.

## Publisher's Note

All claims expressed in this article are solely those of the authors and do not necessarily represent those of their affiliated organizations, or those of the publisher, the editors and the reviewers. Any product that may be evaluated in this article, or claim that may be made by its manufacturer, is not guaranteed or endorsed by the publisher.
